# Fever Nurses: Their Dangers and Defences

**Published:** 1887-05-07

**Authors:** 


### FEVER NURSES:

### THEIR DANGERS AND DEFENCES.

A FEW years ago a discussion on typhoid fever took place at one of the largest of the London hospitals, and almost every physician of eminence in the metropolis attended. The presence of so many severely scientific men restrained the tongues of the garrulous, and kept in check the wayward imagination of those whose speculative tendency was more marked than their practical wisdom. Nevertheless there was no little variety of opinion, especially on the subject of treatment. After listening with much patience, "a potent, grave, and reverend senior" of high scientific distinction took up his parable, and in a few brief words defined his position with regard to the main question. "If," said he, "I had the misfortune to be attacked by typhoid fever, the first and principal thing I should wish for would be an experienced, intelligent, and conscientious nurse. If I were obliged to make a choice between such a nurse and a very active, interfering doctor, I should choose the nurse. I say this from no want of professional loyalty, because no man can feel more profoundly loyal than I do. But I wish to express the conviction that in typhoid fever very active treatment is rarely of service, whilst skilful and unwearied nursing is generally the surest way to recovery." This opinion, so clearly and unhesitatingly expressed, was received with general murmurs of assent, and especially by the fathers and elders who were present.

It would not be easy to define with greater precision the actual status of the intelligent fever-nurse. She is practically a doctor in attendance on her patient, whilst the medical man is the visiting physician. On her, more than on him, depends the issues of the case. In saying this we do not wish it to be understood that a medical man is not necessary in typhoid or other fever. On the contrary, a fever case is eminently one in which the daily or even more frequent visits of the family physician are of the first importance. We merely wish to affirm and to make clear that the competent nurse is really "a doctor in charge" in all such cases.

It is well, nay, it is necessary, that the public should clearly understand who they are who really serve it, and where to look for help. It concerns everyone to know the nature of the instruments made use of for any given purpose, in order that they may render the highest and most enduring service. Accomplished fever-nurses are instruments of the finest temper and of the rarest value, and they should be regarded and used according to their worth. It is no common type of mind that can deliberately choose for its life-work an occupation which daily exposes the worker to imminent risk of rapid and fatal disease. No one knows better than the nurse the dangerous character of those terrible fevers amidst which her days are spent. She sees and realises, as others can not, the dreadful nature of the malady with which her patients are stricken, and the utter futility of all treatment in a considerable proportion of her cases. Of her it may be truly said that she "takes her life in her hand." No Balaclava charge or other deed famous in history demanded more true courage than the unknown and unsung fever-nurse displays every day of her life. To those who really consider these things it is a daily astonishment that so large an army of brave women are constantly ready to face death in the discharge of mere homely duty, which brings neither fame nor substantial reward. In the day of account, when human actions and lives are appraised according to their true value, it need not be doubted that many a duty-loving nurse will hear a "well done" which kings and statesmen might envy.

We are more than pleased to know that all nurses, and especially those who regularly attend fever cases, or who are engaged at fever hospitals, are steadily rising in public estimation. There is yet very much to be desired in regard to the management of the nursing department in many hospitals; and perhaps still more in the way in which nurses are considered in private families. But Rome was not built in a day, and it will no doubt require some time and a large increase of knowledge, before we shall all learn what is fitting and right to be done with those brave soldiers who fight and subdue the enemies which threaten our lives at home. Some time ago we discussed in these columns the question of nurses' recreations. The Belvidere Fever Hospital at Glasgow is about to furnish a practical commentary on the subject. In that huge abode of fever there is accommodation for eighty-two nurses, and the full number is often required. Eighty-two women engaged in one house in the melancholy and dangerous occupation of nursing and caring for five hundred persons in all degrees of dangerous fever! How do the nurses preserve themselves in health or in the necessary courage for their arduous and painful duties? It is proposed by the Chairman, Mr. W. R. W. Smith, to found a library, and the proposal makes one wonder how it is that every fever and other hospital is not already provided with an abundant library of amusing and instructive books. How do nurses occupy their hours of leisure if they do not read? A piano also is wanted. One piano! There should be half-a-dozen. Some time ago there was an outcry about a piano being introduced into a certain hospital. It would disturb the patients, said some; and besides, what do nurses want with pianos and music? All such miserable grumblers ought to experience the comforts of a long and dangerous illness, with nobody but the very fattest and most whiskey-loving of Sairey Gamps to nurse them. A piano, or half-a-dozen of them, we hope the Belvidere managers will purchase without delay. Not only so, but they should teach every nurse in the place to sing and dance, and give them frequent opportunities of exercising those pleasant and health-giving accomplishments.

But Mr. Smith will not be satisfied with a library and a piano for the nurses over whom he presides. He wants a covered tennis-court as well. O rare Mr. Smith! You are as wise as you are doubtless kind. A tennis-court by all means, and a covered one too. Give your nurses the opportunity of using their muscles and expanding their chests, of quickening their circulation and forgetting in the delightful exhilaration of healthful play the depressing and melancholy labours which they daily perform. It may be hoped the committee at the Belvidere will not let the grass grow under their feet. If the good work is to be done, it were well that it be done quickly. We should like to see it accomplished at once, and to hear that it is as successful as its wise and benevolent projector desires. If it should prove so—and there need not be the least doubt that it will—we shall consider it an urgent part of our duty to leave no stone unturned until every other large hospital in the kingdom has abundant means of healthy and cheerful amusement and instruction for its nursing staff. They who give themselves to fever hospitals offer their lives on the altar of duty. The public will be utterly unworthy of such noble self-denial if it does not supply them liberally with everything which can make life useful, healthy, and happy.

